# Beyond CDK4/6 Inhibition: Current Strategies in Hormone Receptor-Positive Metastatic Breast Cancer

**DOI:** 10.1007/s11864-026-01391-3

**Published:** 2026-04-18

**Authors:** Bulent Cetin, Dilek Erdem, Irem Karaman, Ozge Gumusay

**Affiliations:** 1 Department of Internal Medicine, Division of Medical Oncology, Faculty of Medicine, Ondokuz Mayıs University, Samsun, 55280 Turkey; 2 Department of Internal Medicine, Division of Medical Oncology, İstinye University Faculty of Medicine, İstanbul, Turkey; 3 Department of Medicine, Sylvester Comprehensive Cancer Center, University of Miami Miller School of Medicine, Miami, USA; 4 School of Medicine, Department of Medical Oncology, Acibadem University, Istanbul, Turkey

**Keywords:** Hormone receptor positive, Targeted therapies, Molecular markers, Metastatic breast cancer, Resistance, Combination therapy

## Abstract

We focus our second-line treatment strategy on biomarker-defined resistance mechanisms that emerge after progression on CDK4/6 inhibitors. In our view, ESR1 mutation–mediated resistance constitutes a biologically distinct phenotype of endocrine resistance rather than a simple treatment escape. Therefore, in patients with ESR1-mutated tumors, we strongly favor incorporating next-generation oral selective estrogen receptor degraders (SERDs), given their targeted mechanism, favorable tolerability, and efficacy following CDK4/6 inhibition. For tumors harboring alterations in the PIK3CA–AKT–PTEN pathway, treatment selection is similarly biomarker driven. In patients with PIK3CA mutations, fulvestrant combined with alpelisib remains an effective option, though tolerability concerns often influence long-term adherence; accordingly, we increasingly prefer fulvestrant with capivasertib due to its activity across PIK3CA, AKT1, and PTEN alterations and its more manageable safety profile. When patients exhibit rapid progression, endocrine-independent biology, or aggressive visceral disease, we transition early to antibody–drug conjugates such as trastuzumab deruxtecan or sacituzumab govitecan, which demonstrate superior outcomes compared with conventional chemotherapy in appropriately selected cases. Overall, an individualized, biomarker-driven approach remains central to optimizing therapeutic sequencing in advanced HR+ breast cancer.

## Introduction

The evolution of personalized therapy has progressed through several key milestones: beginning with the introduction of tamoxifen in the late 1970s, followed by the development of CDK4/6 inhibitors in 2015, and most recently culminating in the approval of the first four Food and Drug Administration (FDA)-approved therapies (everolimus, alpelisib, capivasertib, inavolisib) for metastatic hormone receptor-positive (HR+) disease that target specific genomic alterations in individual tumors.

Aromatase inhibitors remain foundational due to their suppression of peripheral estrogen synthesis. Selective estrogen receptor modulators (SERMs) demonstrate tissue-specific estrogen receptor (ER) modulation via competitive binding. More recently developed agents include selective estrogen receptor degraders (SERDs) and proteolysis-targeting chimeras (PROTACs), which promote ER protein degradation through distinct mechanisms. The therapeutic pipeline has further expanded to include novel classes such as complete ER antagonists (CERANs) and selective ER covalent antagonists (SERCAs), which irreversibly bind and functionally inactivate the ER through covalent modification [1].

In first-line therapy for endocrine sensitive disease, an aromatase inhibitor combined with a CDK4/6 inhibitor is recommended. In endocrine-resistant settings, fulvestrant paired with a CDK4/6 inhibitor represents the standard approach, with the addition of inavolisib specifically recommended for PIK3CA-mutated tumors. Second-line treatment selection is biomarker-directed: PIK3CA alterations warrant PI3Kα inhibitors (e.g., alpelisib) combined with endocrine therapy, AKT/PTEN pathway abnormalities support the use of AKT inhibitors (e.g., ipatasertib) with fulvestrant, and ESR1 mutations indicate oral SERDs (e.g., elacestrant) or other fulvestrant-based regimens. In the treatment of HR+ breast cancer refractory to endocrine and targeted therapies, the current therapeutic arsenal primarily comprises cytotoxic chemotherapy and antibody-drug conjugates (ADCs). Notably, three FDA-approved ADCs—trastuzumab deruxtecan (T-DXd), sacituzumab govitecan (SG), and datopotamab deruxtecan (Dato-DXd)—have demonstrated superior efficacy over conventional chemotherapy, with significant improvements in both progression-free survival (PFS) and overall survival (OS) in clinical trials.

## Precision Medicine in Oncology: Optimizing Targeted and Endocrine Therapy Combinations with Molecular Markers

The optimization of targeted therapies with molecular markers represents the fulfillment of two fundamental promises in modern medicine: personalized therapy and precision oncology. Central to this paradigm is the principle of delivering “the right drug to the right patient at the right time.” All patients with HR-positive/HER2-negative metastatic breast cancer should be tested for ESR1 mutations; novel oral SERDs demonstrate activity in this population. Multiple agents targeting elements of the PI3K signaling pathway are currently available in clinic, in combination with antiestrogen therapy, for patients with specific mutations (PIK3CA, AKT, PTEN) (Table 1). Predicting response to novel hormonal and targeted therapies may be possible through multigene signatures and emerging sequencing technologies.

**Table 1:** Phase III studies of emerging PI3K/AKT/mTOR pathway inhibitors and novel estrogen receptor-targeting therapies in hormone receptor-positive MBC

Clinical trial	Sample size	Key inclusion criteria	Stratification factors	Primary endpoint
EMERALD: Elacestrant vs. Control Prior chemotherapy: 20% vs. 24% ESR1m: 48% vs. 47% Two prior lines of ET: 46% vs. 41%	466	Postmenopausal women and men with ER+/HER2-advanced or metastatic breast cancer 1 ≤ lines prior chemo for mBC 1 to 2 lines of ET, and documented PD on CDK4 and 6 inhibitor	ESR1 mutation status (by ctDNA) Prior fulvestrant Any visceral disease	Median PFS improvements: ITT: 1.94 > 2.79 m (HR (95%Cl) 068 (0.52–0.90), *p* = 0.0049) ESR1m: 1.87 > 3.78 m; (HR (95%Cl) 0.50 (0.34–0.74), *p* = 0.0005)
EMBER3: Imlunestrant vs. Control No prior chemotherapy, 1 prior line of ET ~ 40% of patients were CDK4/6i-naive ESR1m: 41.7% vs. 35.8%	874	Locally advanced or metastatic ER+/HER2-breast cancer Prior treatment with an AI, alone or in combination with a CDK4/6 inhibitor	Prior CDK4/6i therapy Visceral metastases Region	Median PFS improvements: ITT: 5.5 > 5.6 m; HR (95%Cl) 0.87 (0.72–1.04), p:0.12 ESR1m: 3.8 > 5.5 m; Restricted mean survival diff HR (95%Cl) 2.6 m (1.2–3.9), *p* < 0.001
SOLAR-1: Alpesilib+fulvestrant vs. Fulvestrant	572	Men or postmenopausal women with HR+, HER2-ABC Recurrence/progression on/after prior AI-based therapy Identified PIK3CA status (in archival or fresh tumour tissue)	Stratified by presence of liver/lung metastases and prior CDK4/6 inhibitor treatment	Median PFS: 5.7 m > 11.0 m (HR: 0.65 95%Cl 0.50–0.85, *p* < 0.001
INAVO120:Inavolisib+palbociclib+fulvestrant vs. Plasebo+palbociclib+fulvestrant	325	PIK3CA-mutated, HR+, HER2-ABC by central ctDNA or local tissue/ctDNA test Progression during/within 12 months of adjuvant ET completion	Visceral disease (yes vs. no) Endocrine resistance (primary vs. secondary) Region (North America /Western Europe vs. Asia vs. Other)	Median PFS: 7.3 m > 17.2 m HR 0.42 (95% Cl 0.32–0.55), *p* < 0.001
Capitello-291: Capivasertib+fulvestrant vs. Placebo+fulvestrant	708	Men and pre-/post-menopausal women Recurrence while on or < 12 months from end of adjuvant AI, or progression while on prior AI for ABC ≤ 2 lines of prior endocrine therapy for ABC ≤ 1 lines of chemotherapy for ABC Prior CDK4/6 inhibition allowed	Liver metastases Previous CKD4/6 Geographic region	Median PFS: 3.6 m > 7.2 m HR:0.60 (95Cl 0.51–0.71), *p* < 0.001
VERITAC-2: Vepdegestrant vs. Fulvestrant	624	ER +/HER2- advanced or metastatic breast cancer 1 line of CDK4/6i + ET ≤ 1 additional ET Most recent ET for ≥ 6 months No prior SERD (e.g., fulvestrant, elacestrant) No prior chemotherapy for advanced or metastatic disease	ESR1 mutation Visceral disease	Median PFS:5.0 m > 2.1 m HR:0.57 (0.42–0.77) *p* < 0.001
CCTG MA.40/FINER: Ipatasertib+Fulvestrant vs. Placebo+fulvestrant	250	Stage IV ER+/HER-2 negative breast cancer Prior line of systemic treatment with CDK 4/6i and AI	PIK3CA/PTEN/AKT altered vs. wild type/unknown Primary versus secondary endocrine resistance	Median PFS: 5.32 > 1.94 h:0.61 (0.46–0.81) *p* < 0.0007 AKT pathway altered:5.45 > 1.91 h:0.47 (0.31–0.72) *p* < 0.0005
SERENA-6: Camizestrant+continuing CDK4/6i+placebo for AI vs. Continuing AI+CDK4/6i+placebo for camizestrant	315	Female/male patients with ER+/HER2-ABC All patients that have received AI+CDK4/6i) as initial endocrine-based therapy for ABC for at least 6 months ESR1m detected in ctDNA with no evidence of disease progression	Visceral vs. non-visceral ESR1m detected at first test vs. at a subsequent test Time from initiation of AI+CDK4/6i to randomization: <18 vs. > 18 months Palbociclib vs. ribociclib vs. abemaciclib	Median PFS: 16.0 > 9.2 h: 0.44 (0.31–0.60) *p* < 0.00001

ABC, advanced breast cancer; AI, aromatase inhibitor; CDK4/6i, cyclin-dependent kinase 4/6 inhibitors; ctDNA, circulating tumor DNA; ET, endocrine therapy; ER+, estrogen receptor- positive; ESR1mut, ESR1 mutation; HER2-, HER2-negative; ITT, intention-to-treat; MBC, metastatic breast cancer; PFS, progression-free survival; PIK3CA, phosphatidylinositol-4,5-bisphosphate 3-kinase catalytic subunit alpha; PTEN, phosphatase and tensin homolog; SERDs, selective estrogen receptor degraders

As truncal oncogenic mutations, PIK3CA mutations are well-established drivers that frequently occur as early molecular events, typically detectable at the time of initial metastatic diagnosis or even in primary tumor specimens. ESR1 mutations, which alter the estrogen receptor-encoding gene, exhibit a distinct evolutionary pattern: they are infrequent in treatment-naïve primary breast cancers (< 5% prevalence) but emerge under the selective pressure of aromatase inhibitor therapy. These acquired mutations demonstrate substantial clonal expansion in later-line settings, reaching detectable frequencies in up to 40% of patients with metastatic HR+ disease following second- or third-line treatment [2, 3]. These mutations predominantly localize to the ligand-binding domain of the estrogen receptor, conferring constitutive, ligand-independent activation. This structural alteration mediates resistance to aromatase inhibitors by bypassing their therapeutic mechanism of estrogen deprivation.

The phase III EMERALD trial evaluated elacestrant, an oral SERD, versus standard endocrine therapy in postmenopausal women and men with ER+/HER2- metastatic breast cancer who had received 1–2 prior lines of endocrine therapy, including mandatory pretreatment with CDK4/6 inhibitors [4]. This heavily pretreated population (≥ 40% with two prior endocrine regimens, ≥ 20% with prior chemotherapy) demonstrated modest improvement in median PFS in the intention-to-treat (ITT) analysis (2.8 vs. 1.9 months; HR 0.70). Enhanced clinical benefit was observed in biomarker-selected subgroups, particularly those with ESR1-mutated tumors (median PFS 3.8 months). Further stratification by duration of prior CDK4/6 inhibitor benefit identified additional responder populations, suggesting potential utility of composite predictive biomarkers. Subgroup analyses revealed that patients deriving ≥ 12 months of benefit from prior CDK4/6 inhibitor therapy achieved particularly robust outcomes with elacestrant monotherapy, with median PFS approaching 9 months in the ESR1-mutated cohort [5]. This clinically meaningful improvement suggests that composite biomarkers—incorporating both ESR1 mutation status and duration of prior CDK4/6 inhibitor response—may optimize patient selection for elacestrant therapy. Based on these efficacy and safety data, the U.S. FDA granted approval to elacestrant for the treatment of ESR1-mutated, endocrine-refractory metastatic breast cancer in January 2023. Current clinical practice guidelines recommend prioritizing its use in patients who demonstrated prolonged (≥ 12 month) benefit from prior CDK4/6 inhibitor therapy, as this subgroup derived the greatest magnitude of clinical benefit in the EMERALD trial (median PFS 8.6 vs. 1.9 months; HR 0.41, 95% CI 0.26–0.63).

The EMBER-3 trial was a prospective, randomized study enrolling men and postmenopausal women with HR+, HER2-negative advanced breast cancer [6]. Eligible patients had either experienced recurrence within 12 months of completing adjuvant therapy with an aromatase inhibitor (AI) plus a CDK4/6 inhibitor or had disease progression on first-line AI therapy with or without prior exposure to CDK4/6 inhibitor in the metastatic setting. Participants were randomized to receive imlunestrant monotherapy, standard-of-care endocrine therapy (fulvestrant or exemestane), or imlunestrant in combination with abemaciclib. This study enrolled a distinct patient population, allowing only one prior line of endocrine therapy and excluding those who had received chemotherapy. Notably, 40% of participants had no prior exposure to CDK4/6 inhibitors. Consistent with findings from the EMERALD trial, imlunestrant demonstrated no significant PFS benefit in the ITT population. However, PFS was significantly improved with imlunestrant monotherapy compared to standard-of-care endocrine therapy in patients harboring *ESR1* mutations, demonstrating an increase from 3.8 months to 5.5 months. Furthermore, the combination of imlunestrant with abemaciclib provided additional clinical benefit over imlunestrant alone, extending median PFS from 5.5 months to 9.4 months, regardless of *ESR1* mutational status. Global health status (GHS)/ quality of life (QOL) and functional domains were maintained across treatment arms in the EMBER-3 trial [7]. Notably, GHS/QOL and functioning were maintained with imlunestrant-abemaciclib combination despite higher rates of patient-reported diarrhea and nausea/vomiting compared to monotherapy.

The present findings compare favorably with those of the concurrent postMONARCH study, which evaluated a similar yet distinct patient population [8]. All enrolled patients in both studies had received prior CDK4/6 inhibitor therapy, predominantly palbociclib. In the postMONARCH trial, the investigational arm assessed the efficacy of fulvestrant in combination with abemaciclib, yielding a median PFS of six months. In contrast, the current study demonstrates a superior median PFS of nine months with the imlunestrant plus abemaciclib regimen.

A critical question arises regarding the efficacy of next-generation oral antiestrogens against specific ESR1 variants. Furthermore, an important consideration is whether dynamic changes in ESR1 mutational burden—assessed through serial liquid biopsies— could serve as a predictive biomarker for treatment response and prognosis. Early-phase data from a study evaluating imlunestrant demonstrate compelling evidence supporting its molecular activity in ESR1-mutated breast cancer [9]. In this analysis, researchers defined molecular response as a ≥ 50% reduction in ESR1 allelic fraction between baseline (cycle 1, day 1) and the first follow-up assessment (cycle 2, day 1). When stratifying approximately 100 patients by this criterion, molecular responders exhibited a markedly superior PFS of nearly 9 months compared to less than 3 months in non-responders. Further examination revealed that approximately 75% of patients experienced reductions in ESR1 allelic burden, while fewer than 20% showed increases. These observations raise important mechanistic questions: Do specific ESR1 variants (e.g., Y537S versus D538G) exhibit differential sensitivity to imlunestrant? Could concurrent genomic alterations, not captured in this analysis, influence treatment response?

The phase I ELAINE 1 study yields clinically significant data regarding the differential activity of lasofoxifene, a novel SERM, compared to fulvestrant in ESR1-mutated breast cancer [10]. This investigation employed allele-specific analysis to evaluate treatment-induced changes in mutant allelic fraction across distinct ESR1 variants (including D538G, Y537S, and others). Notably, lasofoxifene demonstrated superior activity against all examined variants, inducing significantly greater allelic fraction reductions than fulvestrant (*p* < 0.01 for all comparisons). The median reduction in mutant allelic fraction across the entire cohort was markedly higher with lasofoxifene (89.7%; 95% CI: 85.2–93.1) compared to fulvestrant (14.8%; 95% CI: 8.5–21.3). Particularly striking results emerged in the Y537S variant cohort, where fulvestrant treatment was associated with a mean increase of 8.2% (95% CI: 2.1–14.3) in allelic fraction, while lasofoxifene treatment resulted in an 82.4% reduction (95% CI: 76.8–88.0; *p* = 0.003). Not all ESR1 variants are functionally equivalent, as they exhibit distinct biological properties and clinical implications.

PIK3CA mutations are found in ~ 40% of patients with hormone receptor-positive, HER2-negative advanced breast cancer, and are associated with poor prognosis, as well as variable response to PI3K inhibitors [11–17]. Regarding PI3K inhibition, historical data from the SOLAR-1 trial demonstrate that endocrine-refractory patients with PIK3CA-mutated tumors randomized to receive fulvestrant plus alpelisib (vs. fulvestrant alone) showed significant improvement in median PFS, from 5.7 months to 11.0 months (HR 0.65; 95% CI 0.50–0.85; *p* < 0.001) [18].

The INAVO120 trial was a phase III, randomized, double-blind, placebo-controlled study investigating the efficacy of inavolisib in combination with palbociclib and fulvestrant in patients with PIK3CA-mutated, HR+, HER2-negative advanced breast cancer [19]. Eligible participants had measurable disease and had experienced disease progression during or within 12 months of completing adjuvant endocrine therapy. Notably, this represents the first demonsration of statistically significant improvement in OS with a PI3K pathway-targeted drug. The previously reported improvement in PFS was maintained during longer follow-up, and median time to subsequent chemotherapy was substantially delayed by approximately 2 years [19]. The updated PFS analysis demonstrated a median PFS of 7.3 months in the placebo arm compared with 17.2 months in the inavolisib arm, representing a 2-month increase relative to the primary analysis findings [20]. This corresponded to a stratified hazard ratio of 0.42 (95% CI, 0.32–0.55; *p* < 0.001). For the key secondary endpoint of OS, the inavolisib arm showed a statistically significant improvement with a median OS of 34 months versus 27 months in the placebo arm (stratified HR = 0.67; 95% CI, 0.48–0.94). This 7-month absolute difference translated to a 33% relative reduction in mortality risk (*p* = 0.019), crossing the pre-specified statistical significance boundary. In the placebo arm, 72% of patients received second-line chemotherapy, compared with 55% in the inavolisib arm. This difference will be evaluated further in subsequent analyses. The addition of inavolisib demonstrated significantly improved efficacy outcomes compared to placebo. The objective response rate (ORR) increased substantially to 62.7% in the inavolisib arm, representing a clinically meaningful enhancement in antitumor activity. Furthermore, the duration of response (DoR) was markedly prolonged, with the median DoR extending from 11.1 months in the placebo group to 19.2 months in the inavolisib-treated patients, reflecting a 73% improvement in response durability. The safety analysis revealed a higher incidence of serious adverse events (AEs) in the inavolisib arm (27.3%) compared to the placebo group (13.5%), though treatment discontinuation due to adverse events remained relatively low at 6.8% for inavolisib-treated patients. The safety profile was characterized by expected, on-target adverse events including increased incidence of hyperglycemia (63% all-grade; 6.8% grade 3), stomatitis (55% all-grade; 5.6% grade 3), and ocular symptoms (primarily grade 1–2 dry eye or blurred vision). Importantly, these toxicities proved manageable in most cases, as evidenced by the low treatment discontinuation rate (6.8%) due to adverse events.

The CAPItello-291 trial established capivasertib as the first approved AKT inhibitor for second-line metastatic HR+ breast cancer [21]. In this study, predominantly (though not exclusively) CDK4/6 inhibitor-pretreated patients were randomized to fulvestrant with or without capivasertib. The combination demonstrated clinically significant improvement in median PFS from 3.6 to 7.2 months (HR 0.60; 95% CI 0.51–0.71; *p* < 0.001), leading to its rapid incorporation into clinical practice for AKT pathway-activated tumors. Among the significant toxicities observed are hyperglycemia, diarrhea, mucositis, and rash [18, 19, 21]. While the advent of newer therapeutic agents has led to a reduction in the incidence of grade 3 toxicities, further improvements remain warranted. The MA.40 trial evaluated ipatasertib plus fulvestrant versus fulvestrant alone in patients with HR+, HER2-negative advanced breast cancer who had progressed following prior aromatase inhibitor and CDK4/6 inhibitor therapy [22]. Notably, the study incorporated pre-planned stratification by AKT pathway alteration status, with approximately 45% of enrolled patients harboring tumors with AKT pathway alterations. The trial met its primary endpoint, demonstrating statistically significant improvements in PFS for the ipatasertib-fulvestrant combination compared to fulvestrant monotherapy in both the overall population and the AKT-altered subgroup. Regarding safety, no grade ≥ 3 hyperglycemia events were reported. The incidence of grade 1 hyperglycemia showed an 11% absolute increase in the ipatasertib arm (42%) versus placebo (31%), representing a clinically manageable difference. Several key phase III trials are currently underway (Table 2). The VIKTORIA-1 study is investigating gedatolisib, a next-generation PI3K/mTOR inhibitor, in combination with other agents as part of a triplet therapy regimen. Additionally, the CAPItello-292 trial is evaluating a triple-combination approach consisting of fulvestrant, a CDK4/6 inhibitor, and capivasertib in patients with endocrine-refractory disease. These findings represent promising therapeutic opportunities to advance beyond doublet regimens by incorporating triplet combinations, which may offer improved tolerability and enhanced efficacy.

**Table 2:** Ongoing phase III trials evaluating targeted therapies in metastatic breast cancer

Clinical trial	Sample size	Key inclusion criteria	Stratification factors	Primary endpoint
VIKTORIA-1(NCT05501886): Gedatolisib+pablociclib+fulvestrant vs. Alpesilib+fulvestrant vs. Gedatolisib+fulvestrant	701	Patients with HR+/HER2-ABC who received prior CDK4/6 + AI therapy (2nd or 3rd line)	Patients manually assigned to WT or MT arms based on status of PIK3CA	PFS
CAPItello-292(NCT04862663): Capivasertib+palbociclib+fulvestrant vs. Placebo+palbociclib	700	Endocrine-resistant locally advanced (inoperable) or metastatic HR+/HER-breast cancer		PFS
OPERA-01(NCT06016738): Palazestrant vs.SOC in 2/3L ER+/HER- MBC	510	Prior treatments must include 1–2 prior lines of ET	ESR1 mutation	PFS
ELAINE 3 (NCT05696626): Lasofoxifene plus abemaciclib vs.Fulvestrant plus abemaciclib	400	Women and men ER+/HER2-, locally advanced or metastatic breast cancer Progressed on AI plus palbociclib or ribociclib ≥ 1 ESR1 mutation		PFS
evERA (NCT05306340): Giredestrant + everolimus vs. Endocrine therapy+everolimus	320	ER+/HER2-ABC/mBC Prior ET in combination with CDK4/6i		PFS in ESR1m subgroup and ITT population
ADELA (NCT06382948): Elacestrant+everolimus vs. Elacestrant+placebo	240	Adult men and pre-or post-menopausal women ER (+)/HER2(-) unresectable ABC ESR1 mutation Prior ET in combination with CDK4/6i Patients must have previously received at least 1 and no more than 2 lines of endocrine therapy for advanced breast cancer No prior chemotherapy in the advanced setting	Presence of visceral metastases Duration of prior CDK4/6i-based therapy (≥ 12 months vs. < 12months)	BIRC-PFS
ReDiscover-2 (NCT06982521): RLY-2608 + fulvestrant vs. Capivasertib+fulvestrant in PIK3CAm ER + MBC in 2nd line	540	Adult men and pre-or post-menopausal women ER (+)/HER2(-) unresectable ABC Prior ET in combination with CDK4/6i Patients must have previously received at least 1 and no more than 2 lines of ET in the (neo)adjuvant setting with recurrence on or within 12 months of completion or in the ABC setting		PFS
FourLight-3 (NCT06760637): Atirmociclib+letrazole vs. CDK4/6i+letrazole	1020	ABC/mBC not amenable to surgical resection Previously untreated for ABC/ mBC Documented HR+/HER2- status		PFS by BICR

ABC, advanced breast cancer; AI, aromatase inhibitor; CDK4/6i, cyclin-dependent kinase 4/6 inhibitors; ET, endocrine therapy; ER+, estrogen receptor positive; ESR1, estrogen receptor 1; HER2-, HER2-negative; HR+, hormone receptor-positive; ITT, intention-to-treat; mBC, metastatic breast cancer; MT, mutant; PFS, progression-free survival; WT, wild type

A critical therapeutic challenge lies in determining the optimal approach for patients presenting with multiple actionable targets, particularly concurrent PIK3CA and ESR1 alterations. Emerging clinical evidence is beginning to provide preliminary insights into this complex therapeutic scenario. The current analysis of EMERALD trial data demonstrates that in patients with ≥ 12 months of prior CDK4/6 inhibitor exposure who harbor concurrent ESR1 and PIK3CA mutations, elacestrant maintains a statistically significant efficacy advantage over fulvestrant (median PFS 5–6 months vs. 1–2 months) [5]. However, this represents a clinically meaningful reduction from the > 9 month median PFS observed in the PIK3CA wild-type population, suggesting mutation status may modulate treatment response. Recent findings from the EMBER-3 trial demonstrate that in CDK4/6 inhibitor-pretreated patients harboring both PIK3CA and ESR1 mutations, imlunestrant monotherapy achieved a median PFS survival of approximately 4 months [23]. While the double (imlunestrant+abemaciclib) regimen showed significantly superior efficacy, the absence of fulvestrant control arm data and precise prior CDK4/6 inhibitor exposure duration limits comprehensive interpretation. Nevertheless, these results confirm clinically meaningful activity in this dual-mutant population, albeit potentially reduced compared to anticipated outcomes in PIK3CA wild-type cases.

In the current therapeutic landscape outside clinical trials, the principal options for patients with concurrent ESR1 and PIK3CA mutations consist of either elacestrant monotherapy or fulvestrant combined with alpelisib or capivasertib. This decision requires a comprehensive patient-centered discussion incorporating: (1) comparative toxicity profiles, (2) duration of prior CDK4/6 inhibitor exposure, and (3) disease kinetics. However, several critical knowledge gaps remain warranting retrospective or inverse analysis of existing trial data - particularly from INAVO120 and CAPItello-291 - to address: (i) the efficacy of PI3K/AKT inhibitors specifically in dually-mutated populations, (ii) the influence of polyclonality and specific ESR1/PIK3CA variants, and (iii) the prognostic impact of co-occurring alterations in cyclin E, AKT, or RB pathways. Resolution of these questions is essential for optimizing precision medicine approaches in this molecular subset.

**Fig. 1:**
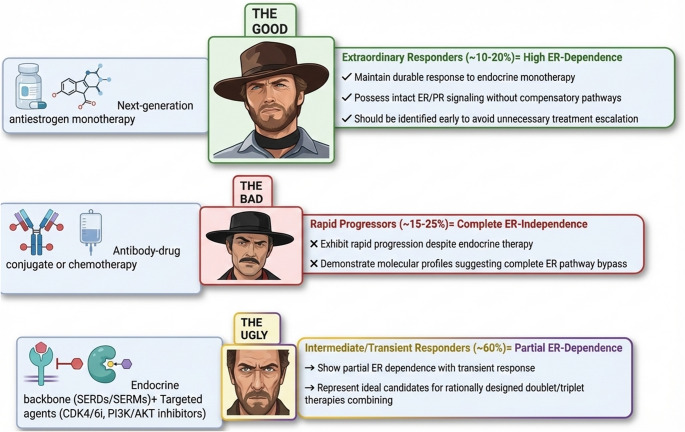
Optimizing therapeutic efficacy through patient population definition. CDK4/6i, cyclin-dependent kinase 4/6 inhibitors; ER, estrogen receptor; SERDs, selective estrogen receptor degraders; selective estrogen receptor modulators SERMs; PI3K, phosphatidylinositol 3-kinase; PR, progesterone receptor

Current advances in molecular profiling are revolutionizing our ability to predict endocrine therapy responsiveness. Through integrated analysis of multigene expression signatures, RNA sequencing data, and epigenetic markers, we can now stratify ER+ breast cancer patients into distinct response categories with clinical implications (Fig. 1): The ER-independent progressor subgroup (approximately 15–25% of cases) demonstrates rapid disease progression despite endocrine intervention, characterized by molecular profiles indicating complete bypass of ER pathway. These patients may derive greater benefit from alternative therapeutic approaches such as ADCs or chemotherapy. Intermediate responders (representing the majority, ~ 60%) exhibit partial ER dependence with transient clinical benefit. This heterogeneous population presents the most significant opportunity for precision medicine interventions, where rationally designed combination therapies incorporating CDK4/6 inhibitors, PI3K/AKT pathway blockers, or novel epigenetic modulators could potentially enhance and prolong treatment responses. A clinically important minority (10–20%) constitutes the exceptional responder cohort, maintaining durable disease control for years with endocrine monotherapy alone. Identification of these patients through comprehensive molecular profiling is particularly valuable, as it could prevent unnecessary treatment escalation and minimize associated toxicities.

Ongoing translational research efforts are employing multigene expression signatures, whole-transcriptome RNA analysis, and comprehensive epigenetic profiling to refine our assessment of estrogen receptor pathway dependence. These approaches promise to transform personalization of care in the second-line setting and beyond. The continued integration of molecular profiling with clinical trial design will be essential for identifying novel actionable targets and developing the next generation of rationally designed therapies.

### Emerging Treatment Strategies for HR+ Breast Cancer in the Second-Line Setting and Beyond

The therapeutic landscape for HR+ metastatic breast cancer after progression on CDK4/6 inhibitors remains challenging, with current options demonstrating limited efficacy. Available strategies in this setting, including PI3K/AKT/mTOR pathway inhibitors and continued CDK4/6 inhibition with endocrine therapy switch, typically yield median PFS of 6–7 months. The oral SERD elacestrant showed modest activity in the EMERALD trial (median PFS 3.8 months overall, 5.5 months in ESR1-mutant patients), while PI3K inhibitor combinations like alpelisib plus fulvestrant achieved median PFS of 7.3 months in PIK3CA-mutated tumors in the BYLieve trial [4, 24]. Recent EMBER-3 trial data presented more encouraging results, with the imlunestrant-abemaciclib combination demonstrating median PFS of approximately 9 months in the post-CDK4/6 inhibitor setting [6].

A fundamental objective in the current therapeutic development for HR+ breast cancer focuses on creating agents that achieve an optimal balance between efficacy and safety. This approach aims to substantially extend the duration of endocrine-sensitive disease control while delaying the need for chemotherapy. Several novel endocrine agents are specifically designed to address three major clinical challenges: treatment-related toxicities associated with current agents, suboptimal pharmacokinetic profiles, and mechanisms of endocrine resistance [25, 26].

The oral SERD camizestrant has emerged as a promising therapeutic option based on results from the phase 2 SERENA-2 trial, which compared camizestrant directly with fulvestrant in fulvestrant-naïve patients with second-line metastatic HR+ breast cancer [27]. The 75 mg daily dose, now advancing to phase 3 evaluation, demonstrated clinically meaningful activity that was particularly enhanced in ESR1-mutant tumors, consistent with the class effect observed with next-generation oral SERDs such as elacestrant and imlunestrant. Camizestrant’s safety profile presents two unique but manageable side effects: low-grade bradycardia (typically asymptomatic) and photopsia (transient visual disturbances), both of which were grade 1–2 in severity and did not lead to treatment discontinuation in the trial. SERENA-6 is the first global registrational phase 3 study to demonsrate the clinical utility of ctDNA monitoring to detect and treat emerging resistance in breast cancer [28]. All patients had been receiving first-line endocrine-based therapy consisting of an aromatase inhibitor plus a CDK4/6 inhibitor (ribociclib, abemaciclib, or palbociclib) for at least six months as their initial treatment for advanced disease. Eligibility for this treatment phase required the detection of ESR1 mutations in ctDNA in the absence of radiographic evidence of disease progression. A total of 315 patients were randomized 1:1 under a double-blind protocol to either continue their current aromatase inhibitor plus CDK4/6 inhibitor therapy along with a matched placebo for camizestrant, or to switch to camizestrant plus the CDK4/6 inhibitor along with a matched placebo for the aromatase inhibitor. ESR1 mutations were identified at the first ctDNA testing timepoint in just over half of the patients. Analysis of the primary endpoint, investigator-assessed PFS, demonstrated a significant improvement with camizestrant. The median PFS was 9.2 months in the control arm (aromatase inhibitor plus CDK4/6 inhibitor) compared to 16.0 months in the experimental arm (camizestrant plus CDK4/6 inhibitor). This difference corresponded to an adjusted hazard ratio of 0.44 (95% CI [0.31–0.60], *p* < 0.001), representing a 56% reduction in the risk of disease progression or death and more than a doubling of median PFS. Camizestrant plus CDK4/6i delayed time to deterioration in quality of life versus continuing aromatase inhibitor plus CDK4/6i, and was well tolerated with a very low rate of treatment discontinuations due to AEs. Notably, only 1% of patients in the camizestrant arm discontinued treatment due to drug-related AEs. A modest increase in the incidence of neutropenia was observed in the camizestrant arm. An analysis of symptomatic adverse events revealed a generally similar profile between the two treatment groups. However, photopsia—characterized by brief flashes of light in the peripheral vision—occurred at a higher incidence in the camizestrant arm (20%) compared to the aromatase inhibitor arm (8%). These events were predominantly grade 1 in severity and did not impact patients’ daily activities. Switching form an aromatase inhibitor to camizestrant with continuation of CDK4/6 inhibitor guided by the emergence of ESR1 mutations during first-line therapy prior to disease progression, significantly improved PFS in patients with HR+, HER2 negative advanced breast cancer.

Vepdegestrant (ARV-471), an oral PROTAC ER degrader, directly binds both E3 ubiquitin ligase and the ER to trigger ubiquitination of ER and its subsequent proteasomal degradation [29, 30]. In contrast, SERDs indirectly recruit the ubiquitin-proteasome system, secondary to conformational changes and/or immobilization of ER.

Phase 2 expansion cohort data demonstrate that the 200 mg oral daily dose (selected for phase 3 evaluation) exhibits enhanced activity in ESR1-mutant tumors, consistent with the mechanistic advantage of targeted ER elimination versus conventional receptor blockade [31]. CBR (rate of confirmed complete response, partial response, or stable disease ≥ 24 weeks) was 37.1% in the overall population and 47.4% in patients with mutant ESR1.Median PFS was 3.5 months (95%Cl: 1.8–8.2) in all evaluable patients and 5.7 months (95%Cl:1.8–8.5) in patients with ESR1 mutations. The safety profile appears favorable, with predominantly grade 1–2 AEs including gastrointestinal disturbances, fatigue, and hot flashes, and no dose-limiting toxicities observed at therapeutic doses.The pivotal VERITAC-2 phase 3 trial represents a critical evaluation of the novel PROTAC agent vepdegestrant (ARV-471) compared to fulvestrant in patients with HR+/HER2- metastatic breast cancer who have progressed after one line of CDK4/6 inhibitor therapy and no more than two lines of prior endocrine treatment [32]. Among patients enrolled in the trial, 43% harbored an ESR1 mutation at baseline. Vepdegestrant demonstrated a statistically significant improvement in median PFS compared to fulvestrant (5.0 months vs. 2.1 months; hazard ratio [HR] = 0.57, *P* **<** 0.01). In the ITT population (regardless of ESR1 mutation status), no significant PFS difference was observed between vepdegestrant (3.7 months) and fulvestrant (3.6 months). Vepdegestrant demonstrated a clinically meaningful improvement in clinical benefit rate **(**CBR) compared to fulvestrant, with rates of 42.1% versus 20.2%, representing a greater than two-fold increase. The treatment effect was even more pronounced in objective response rate (ORR), where vepdegestrant achieved 18.6% compared to fulvestrant’s 4.0%, corresponding to a greater than four-fold improvement. Vepdegestrant demonstrated favorable tolerability in clinical trials, with low rates of treatment discontinuation (3%) and dose reductions (2%). The most frequently reported adverse event was any-grade fatigue, occurring in 27% of patients. Liver function test (LFT) elevations and nausea were the second and third most common adverse effects, with any-grade events remaining in the low teens (≤ 15%). A dedicated QT interval substudy (*n* = 88) demonstrated that vepdegestrant treatment was associated with QT prolongation in 10% of patients.

Unlike SERMs such as tamoxifen, which primarily inhibit the activating function 2 (AF2) domain of the ER, complete estrogen receptor antagonists (CERANs) exert dual suppression by inactivating both the AF1 and AF2 domains. This comprehensive inhibition results in near complete suppression of ER-mediated transcriptional activity. Phase 2 trial results evaluating palazestrant (OP-1250) as monotherapy demonstrate consistent antitumor activity, including in heavily pretreated populations with prior CDK4/6 inhibitor and fulvestrant exposure [33, 34]. Notably, enhanced efficacy has been observed in *ESR1*-mutant tumors, suggesting a potential preferential role in this molecular subset. Regarding safety, the most notable adverse event associated with palazestrant is neutropenia, which requires monitoring in clinical practice. Currently, the registrational phase 3 OPERA-01 trial (NCT06016738) is underway, comparing palazestrant to physician’s choice of endocrine therapy in the second- or third-line metastatic breast cancer setting.

Current clinical evidence demonstrates that oral SERDs and other novel endocrine agents consistently show therapeutic benefit in ESR1-mutant tumors compared to standard endocrine therapy, regardless of overall trial outcome - as seen in positive studies like EMERALD and negative trials such as acelERA (giredestrant) and AMEERA-3 (amcenestrant) [4, 35, 36]. However, critical limitations remain: approximately 40% of patients experience early progression on monotherapy, with nearly identical dropout patterns observed across studies, while 60% derive less than six months of clinical benefit. These findings underscore both the potential of these agents in molecularly-selected populations and the imperative to develop combination strategies that can overcome intrinsic resistance mechanisms and improve durable response rates in advanced breast cancer.

The EMBER-3 trial’s subgroup analysis evaluated imlunestrant plus abemaciclib versus imlunestrant monotherapy across molecular subtypes [6]. In the ESR1-mutant cohort, combination therapy demonstrated superior efficacy with a median PFS of 11.1 months compared to 5.5 months for monotherapy (HR 0.53). Similarly, ESR1 wild-type tumors showed improved outcomes with the combination (median PFS 9.1 vs. 5.5 months). These results demonstrate consistent clinical benefit for the dual-therapy approach regardless of ESR1 mutation status. Similarly, activity has been observed with other novel SERMs, such as lasofoxifene. The ELAINE-2 trial, a small phase 2 study involving 29 patients, demonstrated a promising signal with a median PFS of 13 months [37]. These findings support the rationale for the ongoing registrational Phase 3 trial, ELAINE-3. This study is focused on a second-line metastatic breast cancer population harboring ESR1 mutations, comparing lasofoxifene plus abemaciclib versus fulvestrant plus abemaciclib [38].

Beyond combinations with CDK4/6 inhibitors, emerging data are expected for targeted agents against the PI3K/AKT/mTOR pathway, particularly the mTOR inhibitor everolimus. The ongoing evERA trial (NCT05306340), a phase 3 study, is evaluating giredestrant (an oral selective estrogen receptor degrader, SERD) plus everolimus versus physician’s choice of endocrine therapy plus everolimus. This trial features co-primary endpoints of PFS in both the ESR1-mutant and ITT populations. Results are expected by the end of this year. Similarly, the ADELA trial (NCT 06382948) represents an intriguing investigation, evaluating elacestrant plus everolimus versus elacestrant monotherapy in patients with ESR1-mutant tumors. Results are expected by the end of this year. Similarly, the ADELA trial (NCT 06382948) represents an intriguing investigation, evaluating elacestrant plus everolimus versus elacestrant monotherapy in patients with ESR1-mutant tumors.

Moving to the next emerging class of therapeutics, PI3K inhibitors, several agents have garnered attention, including inavolisib (evaluated in the SOLAR-1 trial) and capivasertib. While head-to-head comparison trials, such as INAVO121 (inavolisib vs. alpelisib), are underway, the field is particularly enthusiastic about next-generation mutant-selective inhibitors. This enthusiasm stems from the dose-limiting toxicities associated with currently approved agents—including hyperglycemia, diarrhea, and rash—which are believed to arise from on-target inhibition of wild-type PI3K [39]. By contrast, newer mutant-selective inhibitors aim to minimize these adverse effects by selectively targeting PI3Kα mutants while sparing wild-type signaling, thereby improving therapeutic tolerability [39].

The next-generation mutant-selective PI3K inhibitors offer significant therapeutic potential through their unique allosteric binding mechanism, which selectively targets mutant isoforms while sparing wild-type PI3K inhibition. This approach addresses the key limitation of current ATP-competitive inhibitors by potentially reducing on-target toxicities. Several promising agents in development include OKI-219, a PI3Kα H1047R-specific allosteric inhibitor; RLY-2608, a novel pan-mutant-selective PI3Kα inhibitor; and additional compounds currently under investigation. Initial results from the phase 1 study evaluating RLY-2608 in combination with fulvestrant demonstrate promising clinical activity, with an ORR of 38% and a median PFS of 9.2 months [40]. The STX-478 monotherapy data demonstrate encouraging clinical activity in the hormone receptor-positive breast cancer cohort, with an observed overall response rate of 23% [41]. A primary focus of these investigations was to determine whether the toxicity profiles of next-generation PI3K inhibitors could demonstrate meaningful improvement over currently approved agents. Comparative analysis of both trials revealed markedly reduced incidence rates of Grade 3/4 adverse events—particularly hyperglycemia, diarrhea, and rash—relative to established therapeutic standards [40, 41]. While these observations originate from limited cohort sizes, the favorable toxicity trends align with preclinical projections for these investigational compounds. Notable safety signals requiring further characterization include modest creatinine elevations, which will be systematically evaluated in expanded patient populations. Similarly, observed transaminase (AST/ALT) elevations warrant careful longitudinal monitoring. These findings have informed the design of the ongoing registrational phase 3 ReDiscover-2 trial (NCT06982521), which employs a randomized, head-to-head comparison of RLY-2608 plus fulvestrant versus capivasertib plus fulvestrant in patients with PIK3CA-mutated, HR+ advanced breast cancer. OKI-219, a mutant-selective PI3Kα inhibitor, has shown preclinical efficacy in PI3Kα-H1047R-mutated models, without the metabolic dysfunction associated with wild-type inhibition, supporting a potential improved therapeutic profile [42]. The collective efficacy and safety data from these next-generation agents—particularly when combined with established endocrine therapies or novel targeted agents—are anticipated to substantially reshape treatment paradigms for PIK3CA-mutated cancers.

As a brief refresher on the classical cell cycle: CDK4/6 regulates the entry into the cell cycle by promoting progression through early G1 phase. CDK2 facilitates the transition from late G1 into S phase, while CDK1 is primarily responsible for driving progression through G2 and entry into mitosis. The classical model of the cell cycle is, in fact, an oversimplification, as multiple cyclin-dependent kinases (CDKs) can functionally compensate for the loss of a single CDK. For instance, CDK4/6, traditionally associated with G1 phase regulation, has also been implicated in S-phase progression. Notably, CDK1 is the only essential CDK required for the initiation of mitosis. The development of selective cell cycle inhibitors is driven by the need to improve therapeutic precision and reduce off-target toxicities [43, 44]. For example, in hormone receptor-positive breast cancers, tumor growth is predominantly dependent on CDK4, with a lesser reliance on CDK6. Notably, treatment-related neutropenia is primarily associated with CDK6 inhibition. Therefore, selective targeting of CDK4 may enhance antitumor efficacy while minimizing hematologic toxicity, offering potential advantages over dual CDK4/6 inhibition. Several CDK4-selective inhibitors are currently in development, with atirmociclib representing the most advanced candidate in clinical evaluation [45]. In the post-progression setting following CDK4/6 inhibitor therapy, atirmociclib demonstrated a disease control rate of 82% and an overall response rate exceeding 30% [46]. In the frontline setting, when combined with letrozole, it also exhibited a favorable overall response rate [47]. Notably, while neutropenia was still observed, its incidence—particularly grade 3 neutropenia (24%)**-**was substantially lower than that associated with CDK4/6 inhibitors. The phase 2 FourLight-1 trial (NCT06105632) assesses therapeutic efficacy in the post-CDK4/6 inhibitor setting, while the phase 3 FourLight-3 trial (NCT06760637) examines a front-line treatment strategy, employing a head-to-head comparison between the experimental regimen and standard-of-care letrozole combined with CDK4/6 inhibition. Is this the optimal path forward? The answer is not so simple. While advancing CDK4-targeted therapies seems logical, emerging evidence suggests that resistance mechanisms—such as compensatory CDK6 overexpression—may undermine efficacy [48, 49]. Therefore, while these trials are essential for elucidating therapeutic potential, exclusive reliance on CDK4 inhibition may not suffice.

*CCNE1* amplification is associated with high levels of cyclin E1 protein, CDK2-dependent proliferation, early progression, and decreased therapy effectiveness [50, 51]. One of the acquired resistance mechanisms is believed to be associated with CDK4/6 inhibition. Clinical validation comes from the PALOMA-3 trial, where patients exhibiting lower baseline cyclin E1 expression derived significantly greater benefit from the palbociclib-fulvestrant combination compared to those with higher expression levels [52]. Additional data presented by Chandralapaty et al. (Memorial Sloan Kettering Cancer Center) demonstrated that p53 pathway alterations correlate with reduced long-term efficacy of first-line CDK4/6 inhibitors in HR+ breast cancer [53]. These findings are derived from the MONALEESA trial data, which highlight a subset of patients with HR+ breast cancer who exhibit diminished long-term response to CDK4/6 inhibition [54, 55]. This suboptimal response may be linked to underlying TP53 mutations, which are observed in approximately 27–30% of HR+ cases, suggesting a potential mechanistic association with resistance.

Targeting CDK2 may restore long-term therapeutic response to CDK4/6 inhibition in patients with HR+ breast cancer, particularly those harboring TP53 alterations. The phase I trial investigating a CDK2 inhibitor in CDK4/6-resistant patients established a recommended phase II dose (RP2D) of 300 mg BID [56]. While the therapy demonstrated preliminary antitumor activity, dose-limiting toxicities were observed, primarily gastrointestinal (with or without concurrent anemia). However, key unresolved challenges persist: (1) the lack of validated biomarkers to identify CDK4/6 inhibitor-resistant populations most likely to benefit, and (2) evidence suggesting that monotherapy may be insufficient due to adaptive feedback mechanisms—including rebound cell-cycle activity—triggered by CDK2 inhibition alone. Combined inhibition of CDK2 abd CDK4/6 promotes durable cell-cycle arrest, highlighting the need for combinatorial strategies in this setting [57]. Multiple CDK2 inhibitors are now in clinical development, including combination regimens. For example, the CDK4/6 inhibitor atirmociclib combined with a CDK2 inhibitor has identified two dose-expansion levels—notably lower than the monotherapy RP2D of 300 mg BID for each agent—highlighting the need for dose adjustments in combinatorial approaches [58, 59]. Future data from these trials are awaited. Efforts to develop a triple CDK2/4/6 inhibitor were limited by a narrow therapeutic window, with unexpectedly low neutropenia despite CDK6 inhibition, suggesting suboptimal target engagement. Further optimization is required to advance this strategy.

Chromatin modulation represents a novel therapeutic strategy to target ER-positive breast cancer. Previous attempts utilizing histone deacetylase (HDAC) inhibitors did not demonstrate significant clinical benefit. An alternative approach could involve the inhibition of histone acetyltransferases (HATs). Lysine acetyltransferases (KAT) play important rugulatory roles in many DNA-dependent processes by acetylating lysine residues on histones. KAT6A/B acetylates lysine residue 23 on histone H3 which leads to an open and transcriptionally active state of chromatin-promoting gene transcription [60]. KAT6 also upregulates genes involved in the cell cycle regulation (E2F1) and promotes MYC signaling and stem cell pathways in breast and other tumor models [61]. KAT6A amplification is associated with poor clinical outcomes in primary breast cancer [62]. Furthermore, KAT6A known to regulate ER expression and silencing leads to downregulation of genes involved in ER signaling, cell cycle, E2F pathway and EMT [63, 64]. The first-in-class KAT6A inhibitor, compound 07248144, was evaluated in a Phase I clinical trial both as monotherapy and in combination with fulvestrant [65]. Monotherapy demonstrated antitumor activity despite a heavily pretreated population (median of five prior lines of therapy), yielding a median PFS of 3 months. In contrast, the combination with fulvestrant (in patients with a median of one prior line of therapy) achieved a median PFS of 10.7 months, suggesting enhanced efficacy in earlier-line settings. Key safety findings included dysgeusia and sporadic grade 3 neutropenia, necessitating close monitoring. Importantly, the combination regimen exhibited encouraging clinical activity in the second- and third-line settings, independent of *ESR1* mutation status or alterations in the PI3K pathway.

The pivotal findings derived from current deliberations indicate a series of significant advancements achieved in the treatment of breast cancer. First, the therapeutic landscape is evolving to include multiple novel endocrine agents with monotherapy potential. Importantly, combination therapies are emerging as a critical strategy and may become the preferred approach moving forward. Among targeted therapies, mutant-selective PI3K inhibitors show particular promise for achieving reduced toxicity while potentially improving clinical outcomes. While CDK2 inhibitors demonstrate limited efficacy as monotherapy, they may have value in combination regimens. Notably, the combination of KAT6 inhibitors with endocrine therapy represents a promising option, particularly given its activity regardless of mutational status following progression on CDK4/6 inhibitors. It is important to recognize that drug development is inherently a long-term process requiring sustained effort. However, through continued collaboration and scientific innovation, we can realistically envision - and work toward - a future where cancer is effectively controlled or eliminated.

## Metastatic HR+ Breast Cancer: the Expanding Role of Cytotoxic Agents—from Chemotherapy to Antibody-Drug Conjugates

ADCs represent a sophisticated class of biopharmaceuticals characterized by three fundamental components: (1) a monoclonal antibody designed for specific antigen targeting on malignant cells, (2) a potent cytotoxic payload, and (3) a specialized linker molecule facilitating stable conjugation between these elements. The structural and functional heterogeneity of these components—including antibody specificity, payload potency, and linker stability—collectively determine the pharmacological profile of each antibody-drug conjugate (ADC), including its target selectivity, therapeutic index, and resistance patterns. The mechanism of ADC activity involves antigen-specific binding followed by receptor-mediated endocytosis, with subsequent trafficking to lysosomes where proteolytic degradation releases the cytotoxic payload. Importantly, certain ADCs exhibit a pharmacologically relevant bystander effect mediated by membrane-permeable payloads that can diffuse out of the target cell.The fundamental pharmacological objective of ADCs is to achieve superior therapeutic index (TI) relative to conventional cytotoxic agents by leveraging target-specific delivery to overcome the narrow therapeutic window characteristic of traditional chemotherapy, wherein the minimum biologically effective dose (BED) frequently converges with the maximum tolerated dose (MTD) [66].

It is important to recognize that all breast cancer cells exhibit some level of HER2 expression [67, 68]. In HER2-positive breast cancer, receptor levels can reach approximately two million per cell. However, even in HER2-normal and HER2-null cases, there may still be around 20,000 receptors present. Immunohistochemistry (IHC) for HER2 was originally developed to identify HER2-positive tumors. The classification becomes more ambiguous in cases of HER2-low expression, typically characterized as IHC 2 + with negative in situ hybridization (ISH) or IHC 1+, and is even more challenging to distinguish in HER2-ultra-low tumors, often classified as IHC 0+ [69–71].

T-DXd represents an innovative ADC incorporating a topoisomerase I inhibitor payload (deruxtecan) with several distinguishing pharmacological characteristics: (1) a high drug-to-antibody ratio (DAR) of 8:1, enabling enhanced cytotoxic payload delivery, and (2) a demonstrated bystander effect that permits activity against heterogeneous tumor populations.The pivotal phase III DESTINY-Breast04 trial (NCT03734029) evaluated T-DXd versus physician’s choice chemotherapy in 557 patients with HER2-low metastatic breast cancer (defined as IHC 1 + or 2+/ISH-) [72]. The study population predominantly consisted of hormone receptor-positive (HR+), endocrine-refractory cases (88.6%) with 1–2 prior chemotherapy regimens. T-DXd demonstrated superior efficacy outcomes, with median progression-free survival (mPFS) doubling from 5.4 months (95% CI, 4.4–7.1) with chemotherapy to 10.1 months (95% CI, 9.5–11.5) with T-DXd (HR 0.51; *p* < 0.001). OS similarly improved from 17.5 to 23.9 months (HR 0.64; *p* = 0.001). Notably, the treatment benefit was consistent across HER2 expression levels, with mPFS maintained at approximately 10 months irrespective of IHC status (10.0 months for IHC 1 + vs. 10.1 months for IHC 2+). The investigation of trastuzumab deruxtecan (T-DXd) in tumors with minimal HER2 expression was explored in the phase III DESTINY-Breast06 trial (NCT04494425), which enrolled chemotherapy-naïve patients with HR+ metastatic breast cancer exhibiting either HER2-low (IHC 1 + or 2+/ISH-) or HER2-ultralow (IHC 0 with faint membrane staining) expression [73]. The study met its primary endpoint, demonstrating superior PFS in the HER2-low population, with median PFS improving from 8.1 months (95% CI 7.1-9.0) with chemotherapy to 13.2 months (95% CI 11.5–14.8) with T-DXd (hazard ratio [HR] 0.62; *p* < 0.0001). Notably, comparable clinical benefit was observed in both the intention-to-treat population (HR 0.63) and HER2-ultralow subgroup (HR 0.69), suggesting therapeutic activity across the HER2 expression continuum. Regarding safety, the most frequent adverse event was nausea (66.3% all-grade; 3.2% grade ≥ 3), while interstitial lung disease/pneumonitis emerged as a critical toxicity (11.2% all-grade; including 1.4% fatal cases), underscoring the need for vigilant monitoring [74]. In the DESTINY-Breast06 biomarker evaluable population, T-DXd demonstrated a greater PFS benefit compared to treatment of physician’s choice, regardless of their baseline PIK3/AKT pathway aberrations, ESR1 mutations or BRCA1 or 2 mutational status [75]. This raises the question of whether HER2-low breast cancer constitutes a distinct biological subtype. Current evidence indicates that there are no definitive genomic differences or prognostic implications associated with HER2-low status [74]. Therefore, the concise conclusion is that HER2-low breast cancer should not be considered a separate biological subtype, but rather a therapeutic classification.

SG is an ADC composed of a TROP2-targeting antibody linked to SN-38, the active metabolite of irinotecan, via a hydrolyzable linker [76, 77]. With a high drug-to-antibody ratio of approximately 8:1, SG demonstrates enhanced tumoricidal activity through two key mechanisms: targeted delivery of its potent topoisomerase I inhibitor payload to TROP2-expressing cells and a pronounced bystander effect mediated by extracellular linker hydrolysis, enabling cytotoxic activity against adjacent tumor cells regardless of TROP2 expression. The phase III TROPICS-02 trial evaluated SG versus standard chemotherapy in heavily pretreated HR+/HER2- metastatic breast cancer patients (median 3 prior therapies) [78]. SG demonstrated statistically significant improvements in both PFS (median 5.5 vs. 4.0 months; HR 0.66, *p* = 0.0003) and OS (median 14.4 vs. 11.2 months; HR 0.79, *p* = 0.02). Notably, prespecified biomarker analyses revealed no differential efficacy based on TROP2 expression levels (interaction p-values > 0.7 for both PFS and OS), indicating that TROP2 immunohistochemical testing is not required for treatment selection. The most frequently reported AEs associated with SG include neutropenia (all-grade: ~60%; grade ≥ 3: ~45%), diarrhea (all-grade: ~65%; grade ≥ 3: ~10%), nausea (all-grade: ~70%; grade ≥ 3: ~5%), and alopecia (~ 40%) [79]. Notably, unlike some other antibody-drug conjugates (e.g., trastuzumab deruxtecan), SG was not associated with interstitial lung disease (ILD) or congestive heart failure (CHF) in clinical trials, suggesting a distinct toxicity profile.

Dato-DXd is a TROP2-targeting antibody-drug conjugate incorporating the same deruxtecan payload as T-DXd but with a lower drug-to-antibody ratio of 4:1 [66]. In the phase III TROPION-Breast01 trial involving patients with HR+/HER2- metastatic breast cancer after 1–2 prior therapies, Dato-DXd demonstrated significantly improved progression-free survival versus chemotherapy (6.9 vs. 4.5 months; HR 0.63, *p* < 0.0001) [80]. While interim overall survival analysis showed no statistically significant difference (HR 0.84, *p* = 0.16), this may reflect confounding from greater subsequent ADC use in the chemotherapy arm, as adjusted analyses suggested a potential survival benefit (19 vs. 17.5 months) [81]. The agent exhibited a favorable safety profile, with unique but manageable ophthalmologic toxicities and stomatitis, low discontinuation rates (3%), minimal grade ≥ 3 ILD (1%), and no reported febrile neutropenia [82, 83].

**Fig. 2:**
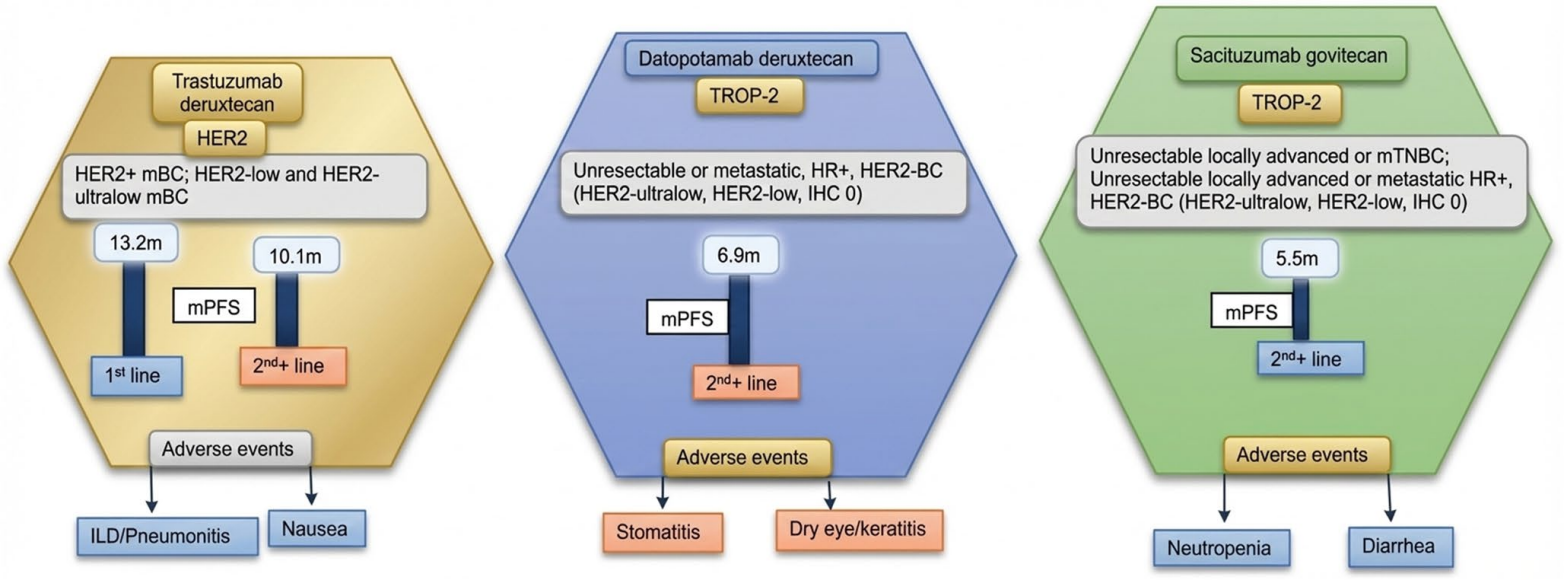
FDA-approved antibody-drug conjugates for hormone receptor-positive metastatic breast cancer. BC, breast cancer; HER2, human epidermal growth factor receptor 2; HR+, hormone receptor-positive; IHC, immunohistochemistry; ILD, interstitial lung disease; mPFS, median progression free survival; mTNBC, metastatic triple-negative breast cancer; TROP-2, trophoblast cell surface antigen 2

How, then, should we sequence these ADCs? While we can consider the adverse event (AE) profiles or dosing schedules, PFS data may not be directly comparable due to significant differences in trial populations. Specifically, the TROPICS-02 study (SG trial) enrolled a more heavily pretreated patient cohort, introducing potential confounding variables when cross-trial comparisons are attempted (Fig. 2).

Many patients are now candidates for multiple ADCs, but optimal strategies for sequencing are unknown [84–86]. Currently, several ongoing clinical trials are specifically evaluating optimal sequencing strategies for ADCs. To optimize ADC sequencing strategies, a critical evaluation of resistance mechanisms is essential. A recent analysis of SG-treated patients with paired pre- and post-treatment tumor biopsies demonstrated the acquisition of concurrent mutations in both *TROP2* and *TOP1* genes [87]. Notably, interrogation of the TCGA database revealed a significant increase in *TOP1* mutation prevalence from < 1% in treatment-naïve metastatic breast cancer cases to 13% following ADC pretreatment. These findings have important therapeutic implications: tumors developing *TROP2* mutations may demonstrate reduced sensitivity to TROP2-directed ADCs, while those acquiring *TOP1* mutations may exhibit resistance to topoisomerase inhibitor-based payloads [88]. Consequently, molecular profiling of these resistance alterations could guide more rational ADC selection—avoiding TROP2-targeting agents in *TROP2*-mutated cases and employing alternative payload strategies for *TOP1*-mutated tumors—thereby enabling a precision medicine approach to overcome ADC resistance mechanisms.

The next generation of ADCs encompasses several innovative platforms, including: (1) bispecific ADCs capable of dual antigen targeting, (2) novel payload classes such as immunomodulatory agents and non-cytotoxic therapeutic modalities, and (3) radioimmunoconjugates incorporating radionuclide payloads [89]. The current therapeutic paradigm for HR+, HER2-negative metastatic breast cancer prioritizes endocrine therapy combined with targeted agents in first- through third-line settings, with agent selection guided by molecular profiling of acquired mutations. However, in cases of endocrine resistance, cytotoxic chemotherapy remains an option, though ADCs have now emerged as superior alternatives. Contemporary data demonstrate that ADCs improve both PFS and OS compared to conventional chemotherapy, while maintaining a comparable - though distinct - toxicity profile that requires careful management.

## Conclusion

**Fig. 3:**
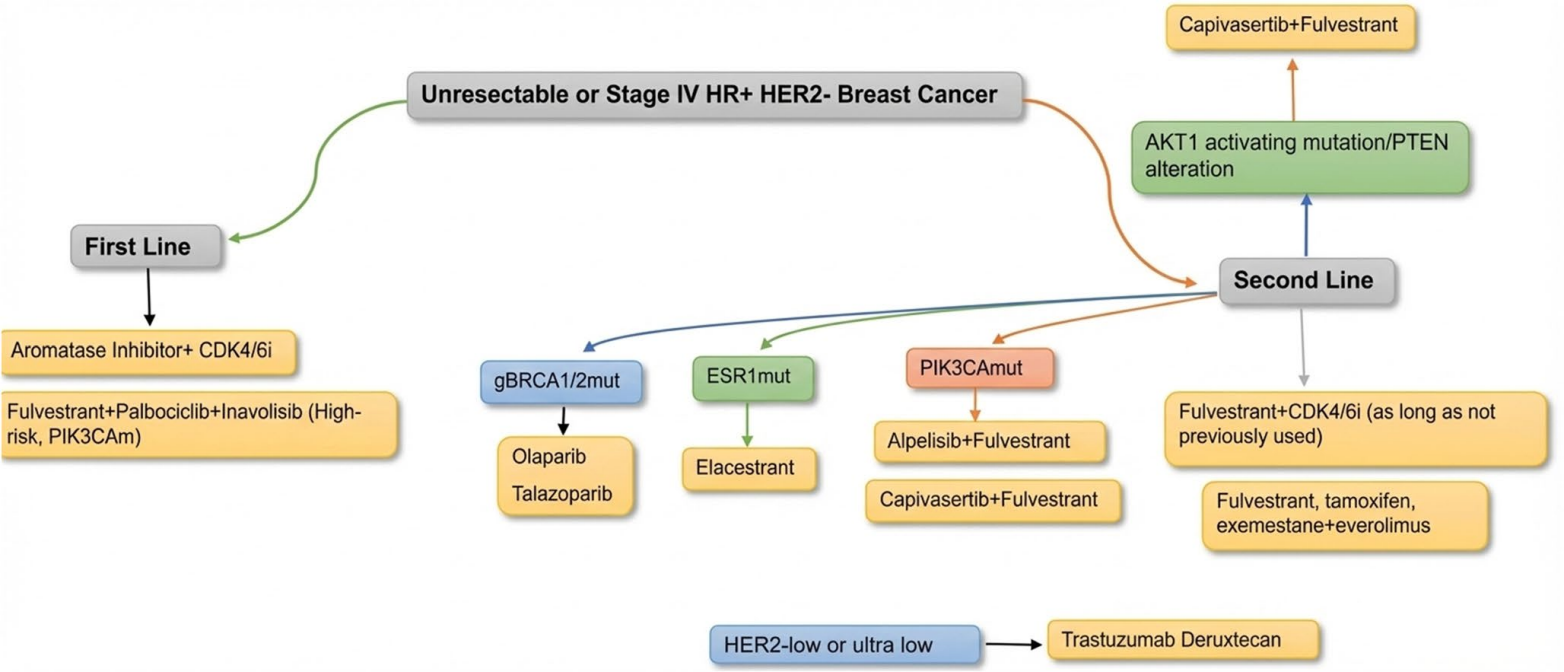
Optimizing treatment strategies for hormone receptor-positive metastatic breast cancer. CDK4/6i, cyclin-dependent kinase 4/6 inhibitors; ESR1mut, ESR1 mutation; gBRCA1/2mut, germline BRCA1/2 mutation; HER2-, HER2-negative; HR+, hormone receptor-positive; PIK3Cam, PIK3CA mutations; PTEN, phosphatase and tensin homolog

The management of HR-positive/HER2 − metastatic breast cancer continues to evolve with the integration of endocrine therapies and targeted agents (Fig. 3). ESR1 mutation testing is recommended for patients with HR-positive/HER2-negative metastatic breast cancer, as novel oral SERDs demonstrate activity in this population. Multiple novel endocrine therapies may gain approval as monotherapies for ESR1-mutant breast cancer, and could become preferred partners for combination with other targeted agents. Multiple agents targeting components of the PI3K signaling pathway are currently available in clinical practice, in combination with antiestrogen therapy, for patients harboring specific mutations, such as PIK3CA, AKT, PTEN. Despite these advances, resistance to endocrine-based therapies inevitably develops in HR-positive metastatic breast cancer. Antibody-drug conjugates including trastuzumab deruxtecan, sacituzumab govitecan, and datopotomab deruxtecan are FDA-approved for the treatment of metastatic HR-positive, HER2-negative breast cancer.

This review has several limitations, including heterogeneity across clinical trial designs and biomarker assessment methods, as well as reliance on evolving sequencing technologies. Furthermore, the lack of mature survival data from many ongoing trials limits the ability to draw definitive conclusions regarding optimal treatment sequencing. Ongoing efforts, including multigene and transcriptional signature anlyses, aim to refine the prediction of ER-dependence and improve personalization of therapy in the second- and third-line metastatic setting. The continued development of predictive biomarkers, coupled with comprehensive correlative analyses from ongoing clinical trials, will be critical to identifying subpopulations with unique molecular features and guiding tailored therapeutic strategies.

## Key References

- Bardia A, Cortés J, Bidard FC, Neven P, Garcia-Sáenz J, Aftimos P, et al. Elacestrant in ER+, HER2- Metastatic Breast Cancer with ESR1-Mutated Tumors: Subgroup Analyses from the Phase III EMERALD Trial by Prior Duration of Endocrine Therapy plus CDK4/6 Inhibitor and in Clinical Subgroups. Clin Cancer Res. 2024;30:4299-309.
  - ○ This article is of outstanding importance because it provides the most robust evidence to date supporting the clinical utility of oral SERDs in ESR1-mutated tumors after progression on CDK4/6 inhibitors.
- Jhaveri KL, Neven P, Casalnuovo ML, Kim SB, Tokunaga E, Aftimos P, et al. Imlunestrant with or without Abemaciclib in Advanced Breast Cancer. N Engl J Med. 2025;392:1189-202.
  - ○ This article is of outstanding importance because EMBER-3 delivers the first phase III evidence for imlunestrant as a next-generation SERD with activity post-CDK4/6 inhibitor therapy.
- Turner NC, Im SA, Saura C, Juric D, Loibl S, Kalinsky K, et al. Inavolisib-Based Therapy in PIK3CA-Mutated Advanced Breast Cancer. N Engl J Med. 2024;391:1584-96.
  - ○ This article is of importance because it introduces inavolisib, a next-generation, mutant-selective PI3Kα inhibitor demonstrating meaningful clinical activity with an improved tolerability profile compared with alpelisib.
- Bardia A, Hu X, Dent R, Yonemori K, Barrios CH, O'Shaughnessy JA, et al. Trastuzumab Deruxtecan after Endocrine Therapy in Metastatic Breast Cancer. N Engl J Med. 2024;391:2110-22.
  - ○ This article is of outstanding importance because it demonstrates that trastuzumab deruxtecan significantly improves outcomes in HER2-low metastatic breast cancer, establishing ADCs as a superior alternative to chemotherapy in selected HR+ disease.
- Turner NC, Oliveira M, Howell SJ, Dalenc F, Cortes J, Gomez Moreno HL, et al. Capivasertib in Hormone Receptor-Positive Advanced Breast Cancer. N Engl J Med. 2023;388:2058-70.
  - ○ This article is of outstanding importance because the CAPItello-291 trial establishes capivasertib plus fulvestrant as an effective and better-tolerated option across PIK3CA, AKT1, and PTEN alterations.

## Data Availability

No datasets were generated or analysed during the current study.
